# A new species of the water mite genus *Sperchon* Kramer, 1877 from China, with identifying *Sperchon rostratus* Lundblad, 1969 through DNA barcoding (Acari, Hydrachnidia, Sperchontidae)

**DOI:** 10.3897/zookeys.707.13493

**Published:** 2017-10-10

**Authors:** Jian-Hua Ding, Jing-Lan Sun, Xu Zhang

**Affiliations:** 1 School of Life Sciences, Huaibei Normal University; Huaibei 235000, China

**Keywords:** China, DNA Barcoding, Hydrachnidia, new species, *Sperchon*

## Abstract

A new species of the water mite genus *Sperchon* Kramer, 1877 from China, *Sperchon fuxiensis* Zhang, **sp. n.**, is described and illustrated in this article. DNA barcoding for the new species is documented for future use. Descriptions of both male and female of *Sperchon rostratus* Lundblad, 1969 are given in the present study, and DNA barcoding for identifying *S. rostratus* is also discussed.

## Introduction

*Sperchon* Kramer, 1877 is the most species-rich genus in the family Sperchontidae Thor, 1900. It is widely distributed in the Holarctic, Oriental, and Ethiopian regions (Cook 1974, Di Sabatino et al. 2008). At present, 22 species of the genus have been recorded from China: *Sperchon beijingensis* Zhang & Jin, 2010; *Sperchon brevipalpis* Jin, 1997; *Sperchon curvipalpis* Zhang & Jin, 2010; *Sperchon fluviatilis* Uchida, 1934; *Sperchon garhwalensis* Kumar, Kumar & Pesic, 2007; *Sperchon gracilipalpis* Lundblad, 1941; *Sperchon heteropoda* Zhang & Jin, 2010; *Sperchon huangshanenses* Zhang & Jin, 2010; *Sperchon lanigerus* Guo & Jin, 2011; *Sperchon mirabilis* (Lundblad, 1941); *Sperchon nikkoensis* Imamura, 1976; *Sperchon oligospinis* Jin, 1997; *Sperchon orbipatella* Zhang & Jin, 2011; *Sperchon perspicuus* Zhang & Jin, 2011; *Sperchon placodermus* (Lundblad, 1967); *S. plumifer* Thor, 1902; *S. rostratus* Lundblad, 1969; *Sperchon sounkyo* Imamura, 1954; *Sperchon synsetus* Zhang & Jin, 2012; *Sperchon turfanensis* Zhang & Jin, 2010; *Sperchon urumqiensis* Zhang & Jin, 2011; and *Sperchon xiaoqikongensis* Zhang & Jin, 2012 (Jin 1997, Zhang et al. 2007, Jin et al. 2010, Zhang & Jin 2010, Zhang et al. 2010, Zhang & Jin 2011, Zhang et al. 2011, Zhang et al. 2012).

Species identification based on the 658bp sequence of mitochondrial cytochrome oxidase I gene (COI)) is known as “DNA barcoding”. This technique has been widely applied in many invertebrates, but rarely in Hydrachnidia (Hebert et al. 2004, Feng et al. 2011, Zhang and Zhang 2014). The study of DNA barcoding for *Sperchon* has not been reported yet.

During checking of a recent collection of water mites, three species (*S. plumifer*, *S. rostratus*, and *Sperchonopsis echphyma* Prasad & Cook, 1972) and a new species (*S. fuxiensis* sp. n.) were found. The descriptions and illustrations of *S. fuxiensis* sp. n. are given herein. DNA barcoding for these four species is also provided. DNA barcoding for indentifying *S. rostratus* is discussed in the present study.

## Materials and methods

Water mites were collected by hand netting and preserved in absolute ethanol in 1.5ml centrifuge tubes. The centrifuge tubes were transported to the laboratory and stored at -20°C. The information of the samples used in this study is given in Table 1.

**Table 1.** Samples examined in this study.

Species	Sex	BOLD process ID	GenBank accession numbers
*Sperchon rostratus*	Male	SPER001-17	MF124260
*Sperchon rostratus*	Male	SPER002-17	MF124259
*Sperchon rostratus*	Female	SPER003-17	MF124258
*Sperchon rostratus*	Female	SPER004-17	MF124257
*Sperchon plumifer*	Female	SPER005-17	MF124256
*Sperchon plumifer*	Male	SPER006-17	MF124255
*Sperchon plumifer*	Male	SPER007-17	MF124254
*Sperchon plumifer*	Male	SPER008-17	MF124253
*Sperchonopsis echphyma*	Male	SPER009-17	MF124252
*Sperchon fuxiensis* sp. n.	Female	SPER010-17	MF124251

### Molecular analysis

For molecular examination, each mite was transferred in individual 1.5ml tubes, and washed several times with sterile deionized water. Non-destructive DNA extraction was done on the whole mite. The genomic DNA was extracted by using the DNeasy Blood & Tissue kit (Qiagen, Hilden, Germany), following the manufacturer’s instructions. Then, the mites were fixed in absolute ethanol and stored at -20°C for morphological analysis.

The standard COI barcoding fragments (658bp) were amplified with the universal primers LCO 1490 (5'-GGTCAACAAATCATAAAGATATTGG-3') and HCO 2198 (5'-TAAACTTCAGGGTGACCAAAAAATCA-3') (Folmer et al. 1994). Primers were synthesized by Shanghai Sangon Biotechnology (Shanghai, China). All amplification reactions were done in a total volume of 25?l, containing 1–5?l DNA; 12.5?l 2?Taq PCR MasterMix (Tiangen, Beijing, China) and deionized water. The PCR amplification was performed with the following profile: 5 min at 94°C;35 cycles of 30 sec at 94°C, 30 sec at 51°C, 45 sec at 72°C; final extension 10 min at 72°C. PCR products were purified by using QIAquick Gel Extraction kit (Qiagen, Hilden, Germany). The pure segments were ligated into the pGEM-Teasy vector (Promega, Madison, WI, USA) and introduced into *Escherichia coli* DH5a cells. Bacteria were cultured in LB medium after blue/white selection, and then inserts were sequenced with M13 primers. Each insert was sequenced twice with ABI 3730 automated DNA sequencer by Shanghai Sangon. All sequences were submitted to BOLD and GenBank. The BOLD process ID and the GenBank accession numbers are provided in Table 1.

All the sequence data were analysed by using MEGA (ver. 6; Tamura et al. 2013), and were aligned by ClustalW. Genetic distances within and between species were calculated with a K2P model. Phylogenetic trees were constructed with neighbur-joining (NJ) and maximum-likelihood (ML) using K2P model. The sequence of *Sperchonopsis echphyma* was used as the outgroup. Bootstrap values were obtained from 1000 replicates.

### Morphometric analysis

For morphological examination, the mite was dissected as described elsewhere (e.g. Cook 1974). Terms follow Jin (1997). The following abbreviations are used:

A1, A2 = antennal glandularia 1 and 2; ACG = anterior coxal group (CxI + CxII); CxI–CxIV = coxae I–IV; D1–D4 = dorsoglandularia 1–4; E1–E4 = epimeroglandularia 1–4; L1–L4 = lateroglandularia l–4; O1, O2 = ocularia l and 2; PCG = posterior coxal group (CxIII + CxIV); P-I–P-V = palpal segments 1–5; V1–V4 = venteroglandularia 1–4; I-L-1–I-L-6 = the first leg segments 1–6; II-L-1–II-L-6 = the second leg segments 1–6; III-L-1–III-L-6 = the third leg segments 1–6; IV-L-1–IV-L-6 = the fourth leg segments 1–6.

The type specimens are deposited in School of Life Sciences, Huaibei Normal University, China. All measurements are given in µm.

## Systematics

### Family Sperchontidae Thor, 1900

#### Genus *Sperchon* Kramer, 1877

##### Sperchon fuxiensis

Taxon classificationAnimaliaORDOFAMILIA

Zhang sp. n.

http://zoobank.org/0B23E95E-F928-4734-AC9C-C0FF6E99F006

Figures 1–3 , 4–9

###### Type series.

Holotype: Female, Anhui Province, Fuxi village, Monkey Valley scenic area, an unnamed stream (30°04'16"N; 118°09'26"E), 8 September 2016, coll. Xu Zhang. Paratypes: 1 female, the same data as the holotype.

###### Diagnosis.

Integument fine spinules arranged in hexagonal pattern; A1 smooth; excretory pore surrounded by a sclerotized ring; P-II with a long ventro-distal projection and one thick seta; third to fifth segments of leg I-IV with short plumose setae.

###### Description.

**Figures 1–3.**
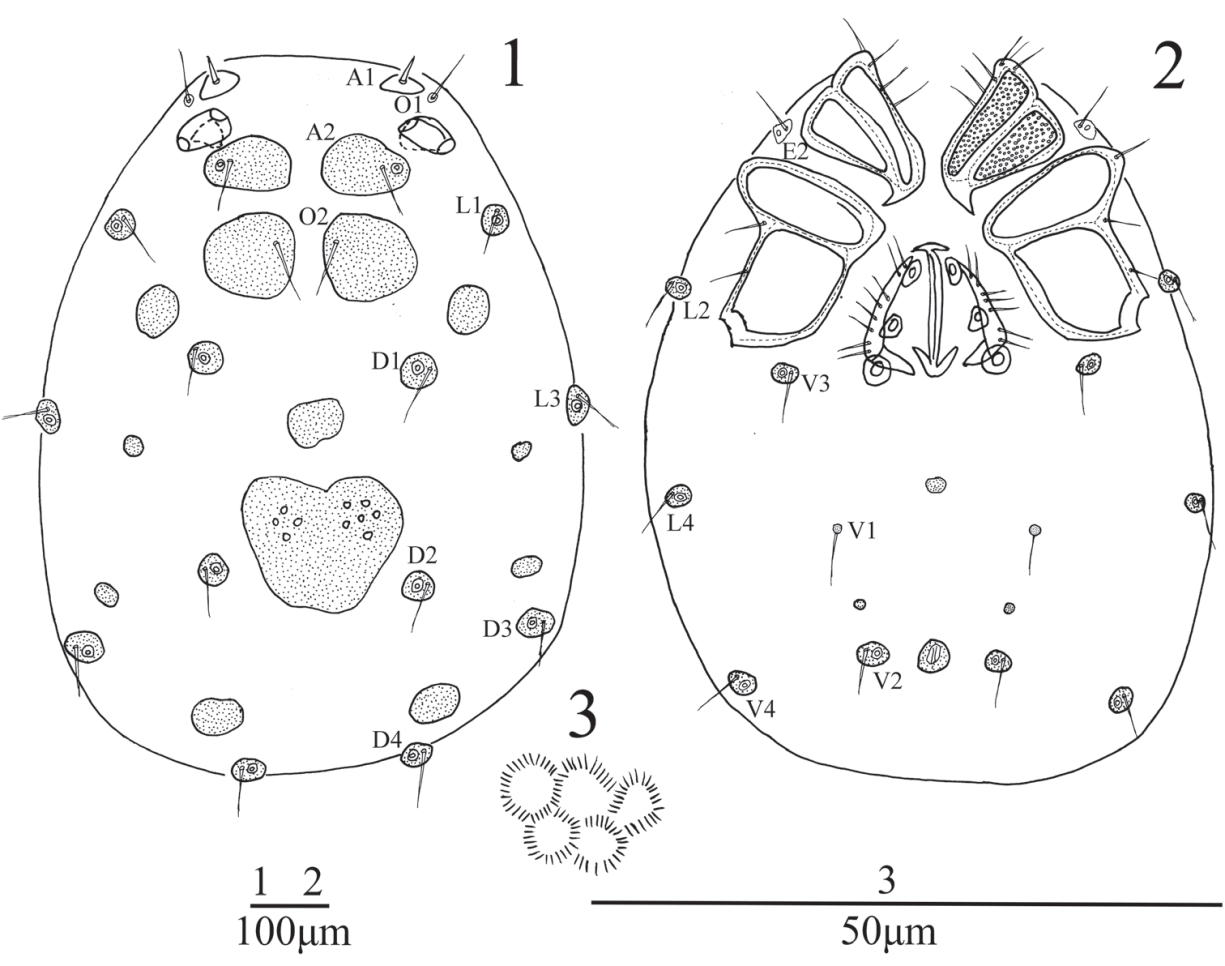
*Sperchon fuxiensis* sp. n., Female **1** idiosoma, dorsal view **2** idiosoma, ventral view **3** structure of integument.

**Female** (n = 2): *Body* oval in shape, 948 (965) in length, 837 (842) in width. Integument yellow in colour, covered with very fine spinules arranged in hexagonal pattern (Fig. 3). A1 short, smooth and thick, other dorsal setae long and thin. Chitinous plates and glandular plates on both dorsum and venter well developed as illustrated in Fig. 1 and Fig. 2. The heart-shaped platelet between D2 somewhat bluish. Coxae in four groups, surface of coxae reticulated. ACG 92 (98) in length, apodeme well developed. E2 laterally between ACG and PCG. PCG 220 (231) in length. E4 absent from CxIII. Distance between anterior end of ACG and posterior end of PCG 373 (380). Genital field 205 (207) in length, 171 (175) in width. Pre- and postgenital sclerites developed. Three pairs of genital acetabula, the first pair of genital acetabula elliptical, the second pair somewhat triangular, and the third pair rounded and larger than the anterior two pairs. V1 on small sclerites and without accompanying glandularia. Excretory pore between V2, and with a sclerotized ring.

**Figures 4–9.**
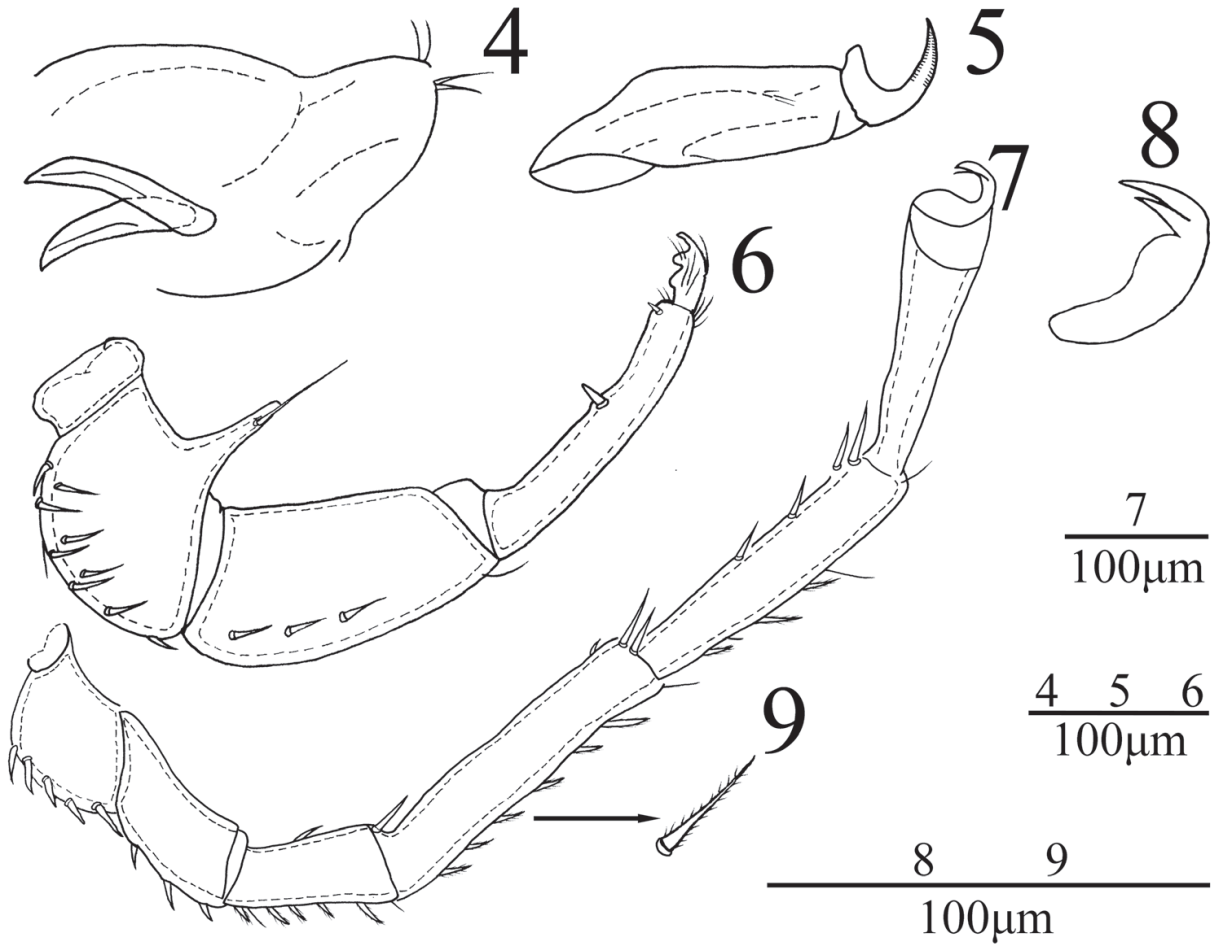
*Sperchon fuxiensis* sp. n., Female **4** infracapitulum **5** chelicera **6** palp **7** IV-L-1–IV-L-6 **8** claw **9** dorsal seta of IV-L-5.

*Capitulum* with a long rostrum, length 213 (219). Chelicera total length 219 (226), basal segment length 158 (164), claw length 61 (62), ratio of basal segment /claw length 2.6. Dorsal lengths of the palpal segments: P-I, 22 (23); P- II, 123 (127); P-III, 172 (178); P-IV, 178 (183); P-V, 36 (37). P-I short and without seta. P-II with a long ventro-distal projection bearing one long setae. Approximately ten setae on the lateral and dorsal side of P-II and none of them plumose. The ventral side of P-III nearly straight and without seta, four short smooth setae on the lateral and dorsal side. P-IV with two small peg-like ventral setae, one larger almost in the middle, another one near the ventral distal end.

*Legs*. Dorsal lengths of leg I: I-L-1, 53 (55); I-L-2, 76 (80); I-L-3, 78 (81); I-L-4, 132 (139); I-L-5, 138 (142); I-L-6, 130 (137). Dorsal lengths of leg IV: IV-L-1, 92 (99); IV-L-2, 126 (135); IV-L-3, 129 (137); IV-L-4, 231 (243); IV-L-5, 225 (231); IV-L-6, 193 (198). Third to fifth segments of leg I-IV with rather short plumose setae in longitudinal rows (Fig. 9). Ambulacrum with two claws. Claws with well protruded claw-blade and two small claws, a long dorsal and a shorter ventral one (Fig. 8).

###### Etymology.

The species is named after the village where it was collected.

###### Remarks.

Due to the shape of the integument, P- II with a very long ventrodistal projection, excretory pore surrounded by sclerotized ring, and third to fifth segments of leg I-IV with plumose setae, the new species is similar to *S. hispidus* Koenike, 1895 and *Sperchon indicus* Kumar, Kumar & Pesic, 2007 (Kumar et al. 2007, Tuzovskij 2010). However, the new species differs from the two species in the shape of the acetabula. In *S. hispidus* and *S. indicus*, three pairs of acetabula are large and arranged densely, whereas in the new species, the three pairs of acetabula are relatively small and arranged loosely with large gaps. The new species also differs from *S. hispidus* and *S. indicus* in the shape of apodemes of anterior coxae, which are indistinct in *S. hispidus* and *S. indicus*, but well developed in the new species. Besides, E4 is situated on CxIII in *S. hispidus* and *S. indicus*, but absent from CxIII in the new species.

###### Distribution.

China (Anhui Province).

##### Sperchon rostratus

Taxon classificationAnimaliaORDOFAMILIA

Lundblad, 1969

Figures 10–12 , 13–17 , 18–19

###### Material examined.

2 females, Guizhou Province, Fanjingshan National Nature Reserve, an unnamed stream (27°54'06"N; 108°36'44"E), 29 July 2001, coll. Jian-Jun Guo; 1 male and 1 female, Guizhou Province, Leigongshan National Nature Reserve, an unnamed stream (26°21'06"N; 108°12'39"E), 3 October 2005, coll. Xu Zhang; 2 male and 5 females, Anhui Province, Fuxi village, an unnamed stream (30°04'16"N; 118°09'26"E), 8 September 2016, coll. Xu Zhang.

###### Description.

**Figures 10–12.**
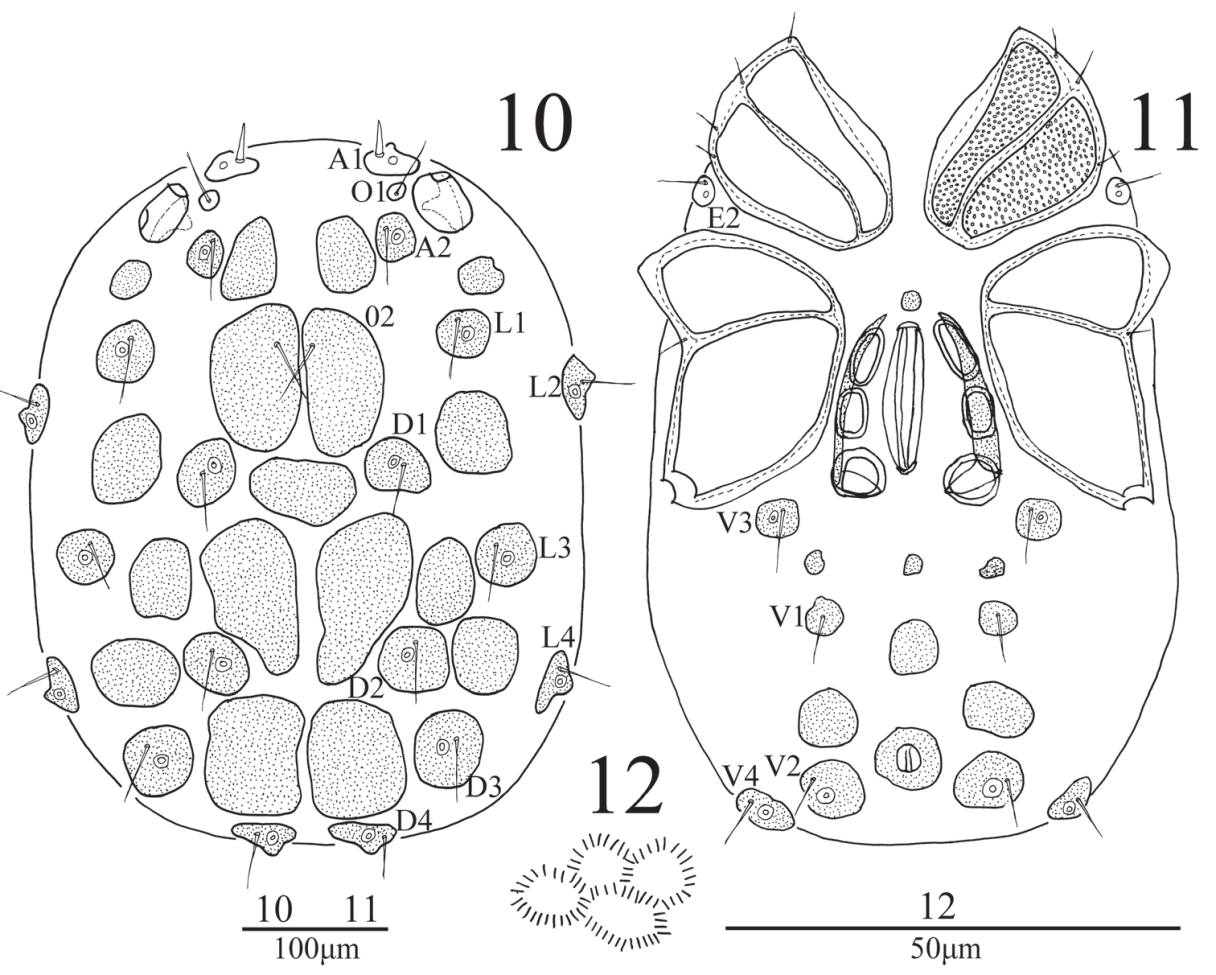
*Sperchon rostratus* Lundblad, 1969, Male **10** idiosoma, dorsal view **11** idiosoma, ventral view **12** structure of integument.

**Male** (n = 3): *Body* oval in shape, 533 (545-576) in length, 432 (441-476) in width, color yellow-brown. Integument with very fine spinules arranged in hexagonal pattern (Fig. 12). Chitinous plates in dorsum and venter well developed as illustrated in Fig. 10 and Fig. 11. All glandularia and O2 surrounded by a platelet. A1 short and smooth, other dorsal setae thin and long. Coxae in four groups, surface of coxae reticulated. ACG 136 (138-152) in length, posterior apodeme indistinct. E2 laterally between ACG and PCG. PCG 194 (204-216) in length, widely separated. E4 absent from CxIII. Distance between anterior end of ACG and posterior end of PCG 329 (347-361). Genital field 135 (142-156) in length, 121 (128-137) in width, with a small and rounded platelet in front. Three pairs of genital acetabula, first and second pairs of acetabula elongate and oval, third pair more or less rounded. Pre- and postgenital sclerite not developed. V1 without accompanying glandularia but on sclerites of medium size. Excretory pore slightly anterior to V2, and surrounded by a well-developed sclerotized ring.

**Figures 13–17.**
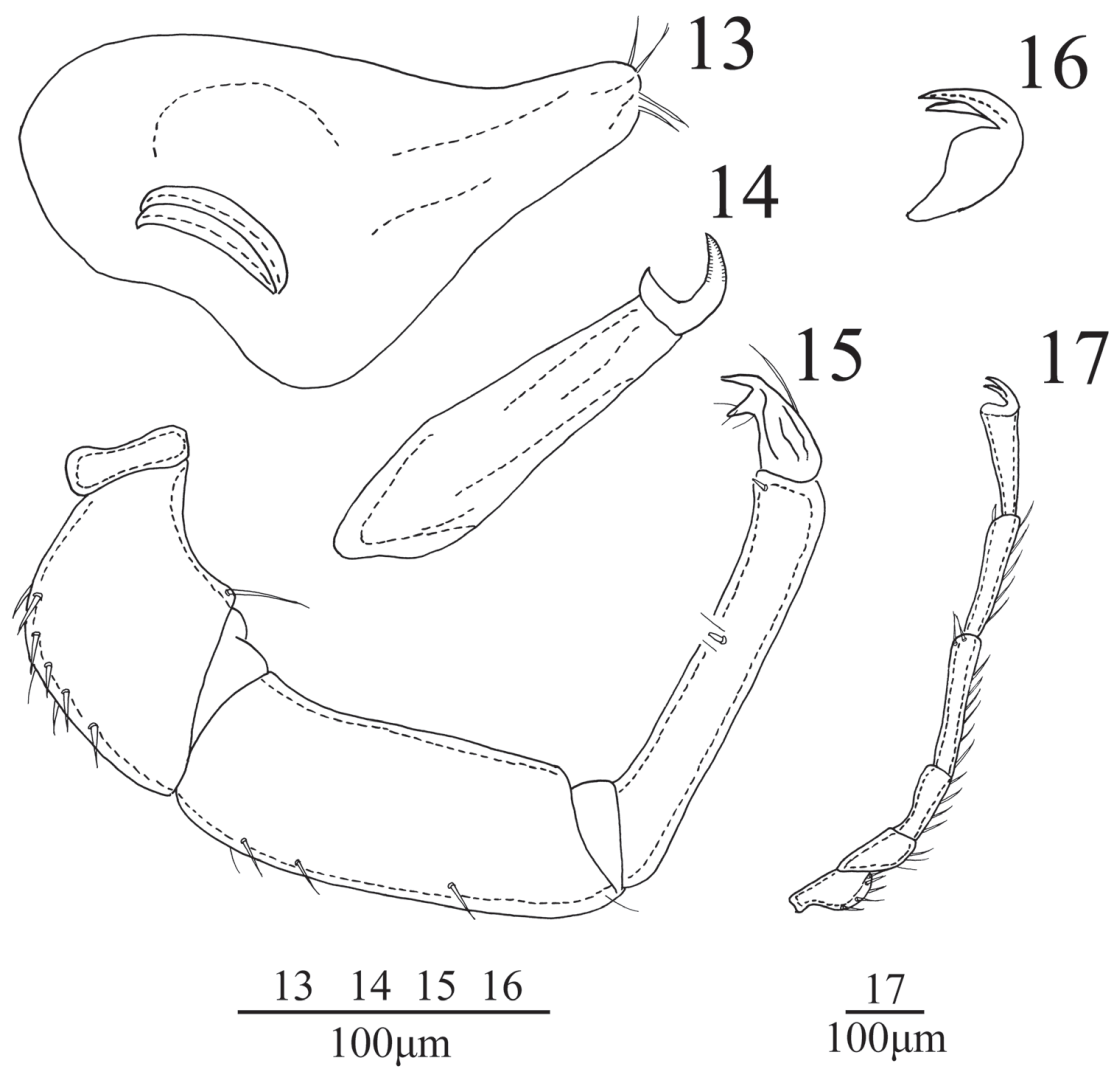
*Sperchon rostratus* Lundblad, 1969, Male **13** infracapitulum **14** chelicera **15** palp **16** claw **17** IV-L-1–IV-L-6.

**Figures 18–19.**
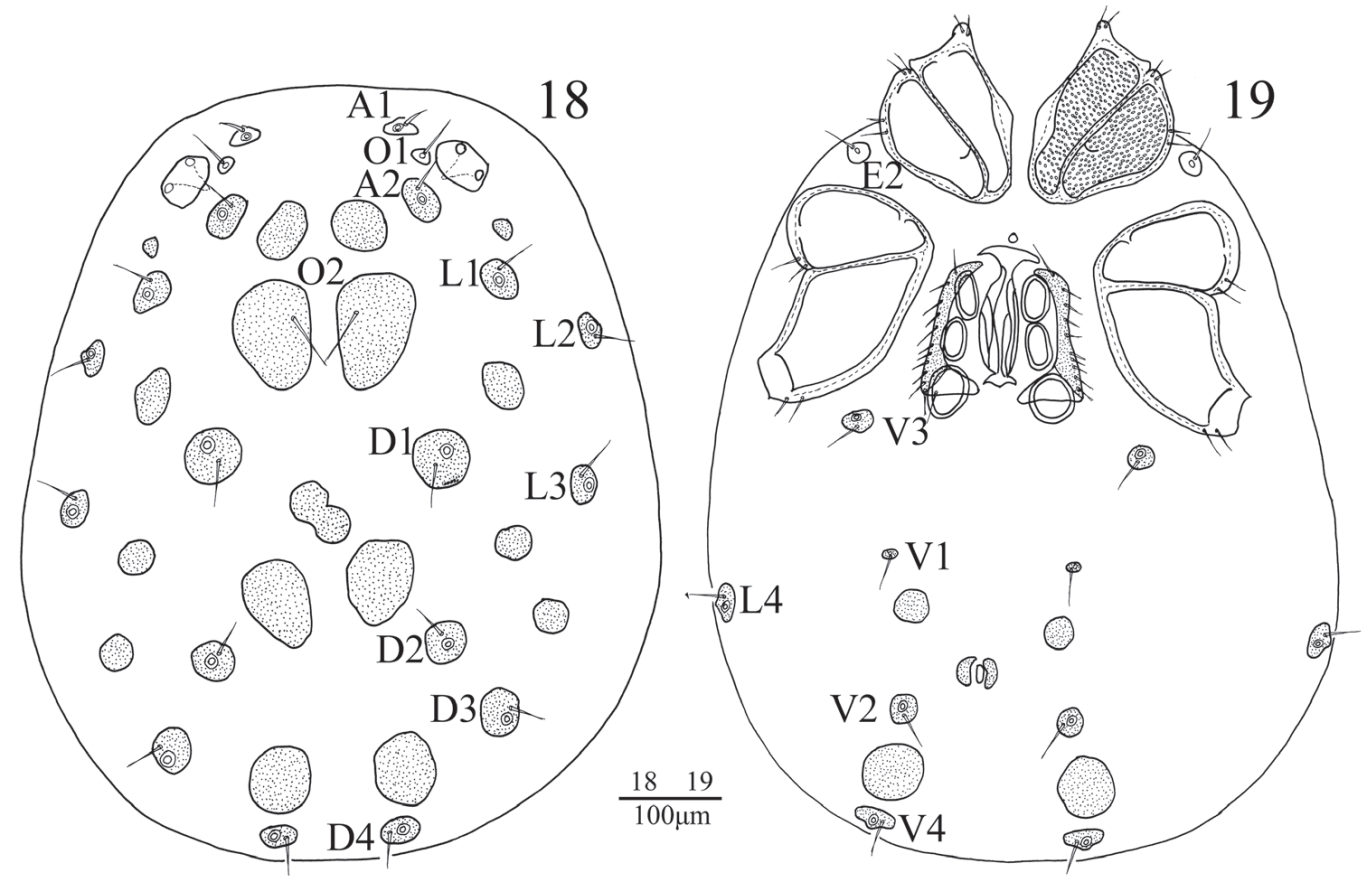
*Sperchon rostratus* Lundblad, 1969, Female **18** idiosoma, dorsal view **19** idiosoma, ventral view.

*Capitulum* with a long rostrum, length 219 (228-236). Chelicera total length 205 (220-227), basal segment length 166 (179-185), claw length 39 (41-42), ratio of basal segment /claw length (4.3-4.4). Dorsal lengths of the palpal segments: P-I, 26 (27-28); P-II, 103 (107-116); P-III, 147 (156-166); P-IV, 152 (161-170); P-V, 36 (39-43). P-I short and without seta. P-II with one thin seta instead of ventro-distal projection. Eight seta on the dorsal and lateral side of the P-II, none of them plumose. The venter margin of P-III without setae, five smooth setae on the lateral and dorsal side. P-IV with two small peg-like setae, one almost in the middle of the segment and with two small setae, another one near the distal end of the segment.

*Legs*. Dorsal lengths of leg I: I-L-1, 41 (44-52); I-L-2, 62 (69-78); I-L-3, 78 (82-94); I-L-4, 86 (89-97); I-L-5, 100 (110-126); I-L-6, 97 (103-117). Dorsal lengths of leg IV: IV-L-1, 76 (82-90); IV-L-2, 83 (92-104); IV-L-3, 107 (113-126); IV-L-4, 113 (124-138); IV-L-5, 175 (192-201); IV-L-6, 152 (165-178). Ambulacrum with two claws. Claws with protruding claw blade and two small claws, a long dorsal claw and a shorter ventral one (Fig. 16).

**Female** (n = 8): Similar to male except for the morphology of genital field and the size of idiosoma. Idiosoma 847 (810-905) in length, 583 (536-618) in width. ACG 173 (154-195) in length, PCG 230 (207-264) in length. Distance between anterior end of ACG and posterior end of PCG 410 (388-435). Genital field 168 (139-192) in length, 152 (138-173) in width. Pregenital sclerite crescent-shaped, and more developed than the postgenital sclerite. Infracapitulum length 288 (264-317). Chelicera total length 286 (278-305), basal segment length 231 (221-248), claw length 55 (57-61), basal segment/claw length ratio 4.2 (4.1-4.4). Dorsal lengths of the palpal segments: P-I, 36 (34-45); P-II, 144 (128-166); P-III, 204 (194-225); P-IV, 212 (200-259); P-V, 57 (50-64). Dorsal lengths of the first leg: I-L-1, 57 (48-66); I-L-2, 86 (71-98); I-L-3, 109 (92-127); I-L-4, 120 (107-146); I-L-5, 142 (130-170); I-L-6, 135 (116-154). Dorsal lengths of the fourth leg: IV-L-1, 93 (80-104); IV-L-2, 112 (107-134); IV-L-3, 173 (157-204); IV-L-4, 296 (272-324); IV-L-5, 267 (257-295); IV-L-6, 234 (227-264).

###### Remarks.

*Sperchon rostratus* was first described from Burma by Lundblad (1969). However, the description and illustration given in the literature are short and insufficient. The species was subsequently recorded from China (Guizhou Province, Taiwan), Iran, and Turkey (Smit 1995, Boyaci 2007, Pesic and Vafaei 2009, Pesic et al. 2012, Pesic et al. 2014). Although the species has been reported many times, an illustration of the idiosoma for the male was given only once (from Turkey) (Boyaci 2007).

Due to the shape of integument, E4 absent from CxIII, P-II with one thin seta, and P-IV with two small peg-like setae, the female from China shows a general conformity with *S. rostratus*, a species previously reported from China, however, the morphological characters of the male show obvious differences between the specimens in our study and the Turkish specimens. It is obvious that the platelets of the dorsum and venter of *S. rostratus* are large and close together (Fig. 10–11), but small and arranged loosely in the Turkish specimens (see details of *S. rostratus* in Boyaci 2007). In addition, the pre- and postgenital sclerites are small in our specimens but relatively large in Turkish specimens, and the pregenital sclerite is somewhat crescent-shaped in Turkish specimens. Additionally, our specimens possess a rounded platelet in front of the genital field, which is absent in the Turkish specimens.

Although there are many differences between the male of *S. rostratus* in our study and the Turkish specimens, considering most characters of our specimens (eg., the shape of integument, E4 absent from CxIII, P-II with one thin seta, P-IV with two small peg-like setae and same habitat of the female), we attribute the male specimens to *S. rostratus*. In order to test whether the male and the female are conspecific, we used DNA barcoding technology for *S. rostratus*. The results are given below (see Results of molecular analysis).

###### Distribution.

Burma, China (Anhui, Guizhou, Taiwan), Turkey, Iran.

### Results of molecular analysis

**Figure 20.**
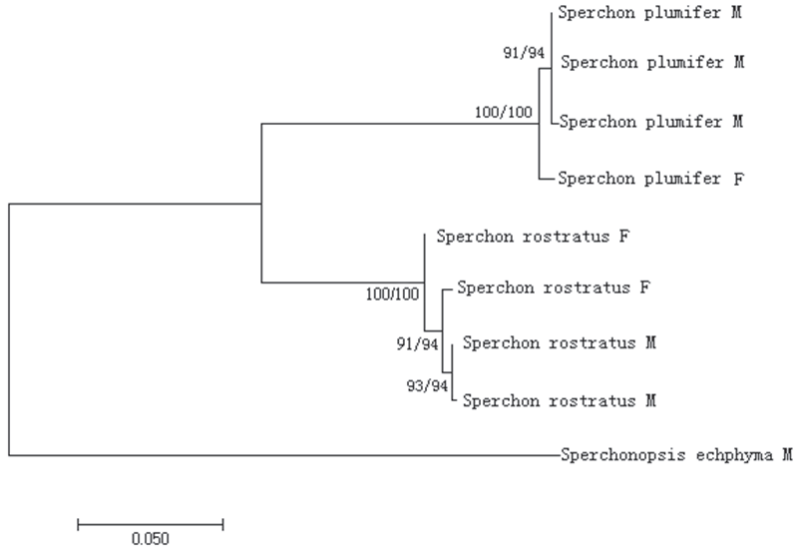
Phylogenetic tree based on barcode region of COI of *Sperchon plumifer*, *Sperchon rostratus*, and *Sperchonopsis echphyma*. The bootstrap proportions of neighbour-joining and maximum-likelihood are indicated above each branch in the format of NJ/ML. *Sperchonopsis echphyma* was used as the outgroup.

The ten nucleotide sequences of 658 bp obtained belong to four species (*S. fuxiensis*, *S. plumifer*, *S. rostratus*, and *Sperchonopsis echphyma*) and two genera (*Sperchon* and *Sperchonopsis*). Sequence of *S. fuxiensis* is documented as DNA barcoding for future use, the others were constructed for a phylogenetic tree and analysed for genetic distances. Phylogenetic tree based on neighbour-joining (NJ) and maximum-likelihood (ML) gave the same result, with minor difference in bootstrap support values only (Figure 20). The male and female of *S. rostratus* were clustered in a clade together with *S. plumifer*.

Genetic distances (K2P) for barcode region of CO I between the species analysed in this study were shown in Table 2. The sequence divergence between the both sexes was 0.6%–1.1% in *S. rostratus*, and 0.9–1.2% in *S. plumifer*, respectively. The intraspecific sequence divergence was 0–1.2% (average is 0.62%) in *S. plumifer*, and 0.2–1.1% (average is 0.73%) in *S. rostratus*, respectively (Table 2). Interspecific divergence between *S. plumifer* and *S. rostratus* was 15.7%–17.1% (average is 16.53%). Intergeneric divergence between *Sperchon* and *Sperchonopsis* was 32.0%–36.6%.

**Table 2.** Genetic distances (K2P) for barcode region of CO I among the *Sperchon* species analysed in this study. (‘F’ indicates female and ‘M’ indicates male).

	1	2	3	4	5	6	7	8
1 *S. plumifer* F								
2 *S. plumifer* M	0.012							
3 *S. plumifer* M	0.009	0.003						
4 *S. plumifer* M	0.009	0.003	0.000					
5 *S. rostratus* M	0.168	0.171	0.167	0.167				
6 *S. rostratus* M	0.170	0.169	0.169	0.169	0.002			
7 *S. rostratus* F	0.166	0.168	0.164	0.164	0.006	0.008		
8 *S. rostratus* F	0.158	0.161	0.157	0.157	0.009	0.011	0.009	
9 *S. echphyma* M	0.364	0.366	0.366	0.366	0.324	0.322	0.322	0.322

## Discussion

In this study, the female of *S. rostratus* coincided with the species as previously reported, but the male specimens showed differences in the size of chitinous plates and genital field. In order to verify whether the male specimens belong to *S. rostratus*, we attempted to use the molecular identification known as “DNA barcoding” to construct a polygenetic tree and analyse genetic distance. Our attribution of the male specimens to *S. rostratus* was supported by molecular data. Phylogenetic tree showed that the male and the female of *S. rostratus* in our study could cluster in the same clade. In addition, the divergence between the male and the female was 0.6%-1.1%, which approximately agrees with the divergence of both sexes of *S. plumifer* (0.9-1.2%).

The morphological characters of the male *S. rostratus* showed obviously differences between China and Turkey. Many characters of the Chinese male specimens in our study, such as with extended and fused chitinous plates in dorsum and venter, pre- and postgenital sclerite weakly developed, and with a rounded platelet in front of the genital field (Figures 10–11), are typical characters of the male sex. On the contrary, most characters of the Turkish male specimens, e.g., with smaller and unfused chitinous plates, the pregenital sclerite is crescent-shaped and without a rounded platelet in front of the genital field, are the typical characters of the female sex (Di Sabatino et al. 2010). In our study, the Chinese male specimens also match the female of *S. rostratus* with the support of molecular data. Therefore, the male specimens of *S. rostratus* reported from Turkey are female and not male. Although *S. rostratus* has been reported many times, the description and illustration are still incomplete. Our study represents the first description and illustration of both sexes.

In recent years, the research of molecular identification and phylogeny have been reported in many genera of water mites, such as *Brachypodopsis*, *Hygrobates*, *Monatractides*, *Neumania*, *Torrenticola*, and *Unionicola* (Ernsting et al. 2010, Pesic et al. 2012, Pesic and Smit 2014, 2016, 2017, Pesic et al. 2017). However, previous research rarely involved molecular analysis of both sexes. Pesic et al. (2012) reported that divergence value between the female and male of *Torrenticola lundbladi* is 0.15% (different in one nucleotide), which is much lower than the divergences in our study (0.6%–1.1% in *S. rostratus* and 0.9–1.2% in *S. plumifer*). However, only one female and one male of *T. lundbladi* were compared in the their study, so that the molecular data is hardly representative.

Hebert et al. (2004) reported that an appropriate threshold for DNA barcoding sequence should certainly be high enough to separate only specimens that very likely belong to different species. Their research also suggested the 10? rule threshold value method, in which interspecific divergences should be nearly ten times higher than intraspecific divergences (Hebert et al. 2004). In our study, the ratio of interspecific divergences to intraspecific divergences in the genus *Sperchon* is 22.64-26.66, which indicates that the DNA barcoding of COI gene may be a useful tool for identifying the *Sperchon* species.

According to the previous research and our study, the intra- and interspecific divergences of water mites were variable among different groups. For example, the intraspecific divergence value was 0.2% in *Torrenticola sabahensis* (Pesic and Smit 2014) and *Brachypodopsis truncata* (Pesic and Smit 2016), 0.3% in *Torrenticola lukai* (Pesic 2012) and *Brachypodopsis crockerensis* (Pesic and Smit 2016), 0.62% in *S. rostratus* (the present study), 1.8% in *Torrenticola kinabaluensis* (Pesic and Smit 2014), and 5.1% in *Torrenticola neoindica* (Pesic and Smit 2014). The interspecific divergence value was 11.6-11.8% in *Torrenticola* (Pesic et al. 2012), 10.7-23.5% in *Brachypodopsis* (Pesic and Smit 2016), 15.7%-17.1% in *Sperchon* (the present study), and 21.8% in *Monatractides* (Pesic and Smit 2014).

The instable divergences among the different species of water mites may be ascribed to limited data and researches on DNA barcoding for water mites. More molecular researches on water mites are required to solve this problem.

## Supplementary Material

XML Treatment for Sperchon fuxiensis

XML Treatment for Sperchon rostratus
